# Microfluidic Fabrication of Hydrocortisone Nanocrystals Coated with Polymeric Stabilisers

**DOI:** 10.3390/mi7120236

**Published:** 2016-12-18

**Authors:** David F. Odetade, Goran T. Vladisavljević

**Affiliations:** Department of Chemical Engineering, Loughborough University, Epinal Way, Loughborough, Leicestershire LE11 3TU, UK; g.vladisavljevic@lboro.ac.uk

**Keywords:** hydrocortisone, drug nanocrystals, nanoprecipitation, microfluidic mixers, solvent-antisolvent crystallization, enteric coating, X-ray diffraction, differential scanning calorimetry

## Abstract

Hydrocortisone (HC) nanocrystals intended for parenteral administration of HC were produced by anti-solvent crystallisation within coaxial assemblies of pulled borosilicate glass capillaries using either co-current flow of aqueous and organic phases or counter-current flow focusing. The organic phase was composed of 7 mg/mL of HC in a 60:40 (*v*/*v*) mixture of ethanol and water and the anti-solvent was milli-Q water. The microfluidic mixers were fabricated with an orifice diameter of the inner capillary ranging from 50 µm to 400 µm and operated at the aqueous to organic phase flow rate ratio ranging from 5 to 25. The size of the nanocrystals decreased with increasing aqueous to organic flow rate ratio. The counter-current flow microfluidic mixers provided smaller nanocrystals than the co-current flow devices under the same conditions and for the same geometry, due to smaller diameter of the organic phase stream in the mixing zone. The Z-average particle size of the drug nanocrystals increased from 210–280 nm to 320–400 nm after coating the nanocrystals with 0.2 wt % aqueous solution of hydroxypropyl methylcellulose (HPMC) in a stirred vial. The differential scanning calorimetry (DSC) and X-ray powder diffraction (XRPD) analyses carried out on the dried nanocrystals stabilized with HPMC, polyvinyl pyrrolidone (PVP), and sodium lauryl sulfate (SLS) were investigated and reported. The degree of crystallinity for the processed sample was lowest for the sample stabilised with HPMC and the highest for the raw HC powder.

## 1. Introduction 

Microfluidic routes have been shown to be highly effective ways of producing nano- and micro-materials, with reduced production costs when compared to previous methods of industrial approach of production of materials, which normally involve the use of large robotic fluidic workstations (often with large space requirements, high cost of labour, high expenses in the final production process, and even right from the research phase). Further innovation of microfluidics was developed, to usher in a scenario of much being achieved for less and importantly, with much lower cost of production [1]. 

Microfluidics is a field of science and engineering dealing with the reactions and processes on a micro-scale level using very small volumes and flow rates of interacting fluids [2]. The high surface area-to-volume ratio of microfluidic channels is highly attractive in many applications, as it offers higher heat and mass transfer rates, reduced residence times [3], and well-controlled and defined process conditions [2,4,5]. Based upon their architecture, microfluidic systems can be divided into planar (2D), such as simple T-, X-, and Y-junctions [6,7], and axisymmetric (3D) [8], which overcome wetting problems of traditional 2D devices and offer higher throughputs. Based on channel geometry and flow patterns, microfluidic systems can be divided into co-flow [9], flow focusing [10], terrace-based [11], junction-based [12] and those combining two or more different geometries, such as co-flow and flow focusing [13]. Microfluidic devices can be constructed from different materials, such as polydimethylsiloxane (PDMS), polymethyl methacrylate (PMMA), glass, single crystal silicon, piezoelectric materials, e.g., lithium niobate, etc.

The microfluidic systems are efficient tools for reliable and reproducible synthesis of micro- and nanoparticles offering many advantages compared to conventional methods [14,15,16]. The advantages of microfluidic devices for the synthesis of micro- and nano- particles include enhanced processing accuracy and efficiency [17], rapid sample turnaround, opportunity for functional integration of many unit operations on a single chip, cost savings from reduced consumption of materials [18], and inherently safer operation due to reduced amounts of hazardous chemicals during synthesis [2,18,19,20]. Microfluidic devices have made possible to continuously vary reaction conditions and add multiple reagents at precise time intervals and locations during the course of reactions [21]. This can be achieved by manipulating the flow rates, temperatures or concentrations of the fluids or varying the configuration of microfluidic channels and flow patterns [22]. 

Microfluidic technology started to converge with nanotechnology [23,24,25] as an emerging platform for fabrication of nanoparticles for drug delivery. Advantages of nanoparticles over conventional drug delivery carriers include the improved stability of active ingredients, enhanced bioavailability of poorly water-soluble drugs [26], targeted delivery [27,28], and especially enhanced accumulation in cancer tissues due to the enhanced permeability and retention (EPR) effect [29]. The other advantages of nanoparticles for drug delivery are longer circulation half-lives, lower systemic drug levels, increased patient comfort, and reduced follow-up care [30]. 

Drug nanocrystals are crystals with a size in the nanometer range composed of 100% drug; there is no excipient as seen in other nanoparticles except a thin layer of stabiliser. Nanocrystals have been shown to be effective forms of drugs because the decrease in the crystal size leads to increase in the surface area-to-volume ratio, which also increases the dissolution rate and saturated solubility of the drug [31], as well as its adhesiveness to cell membranes. They can be produced on a large scale using a variety of top-down or bottom-up methods [32,33], but many of them lack control over the size of nanocrystals. Drug nanocrystals can be used instead of microcrystals for oral bioavailability enhancement, but also suspended in water for intravenous or pulmonary drug delivery [34].

Active pharmaceutical ingredients with poor water solubility cause delivery problems such as poor bioavailability. Techniques such as co-solvent precipitation do not overcome these problems, even though they can solve the solubility issues. This is because such methods do not produce large surface area to ensure the dissolution of the drug at target sites. Nanocrystals improve the saturation solubility of drugs as well as the bioavailability, and are suitable delivery systems for oral intake of drugs with low solubility and poor dissolution and absorption rates. They enhance dissolution rate due to their higher surface energy and surface area, and are also useful due to their low density microstructure [35,36]. Drug nanocrystals can be delivered through various routes of administration such as oral, parenteral and intravenous, dermal, ocular and pulmonary [37,38].

Just as microparticles, nanocrystals also have two broad methods of production. The top-down methods involve disintegration of drug particles by mechanical attrition such as milling and homogenisation, while the bottom-up techniques typically involve antisolvent nanoprecipitation and crystallisation, sonocrystallisation, mixing, sonoprecipitation, etc. [39]. Some of the issues with both the top-down and bottom-up techniques include cost, time, large size distribution of particles, and possibility of re-crystallisation of the nanocrystals produced. Therefore, the decrease in bioavailability, especially in a pharmaceutical product (unlike food) is inevitable and the chemical structure of the drug molecule could also be affected [35,37,40].

Microfluidic devices can allow unprecedented control over crystallisation conditions at the micro-scale thereby allowing better control over the properties such as crystal size and polymorph type, while minimizing the consumption of chemicals and the amount of waste.

Glass capillary microfluidic devices possess a unique blend of properties which make them attractive options for the synthesis of nanoparticles. Glass has superior optical transparency, offers excellent control over surface properties and has a high chemical resistance to acids, alkalis and organic solvents, which gives glass capillary devices an edge over PDMS devices [41]. In addition, glass capillary devices can overcome the surface wetting problems and deposition of the synthesized materials onto the surface of the walls, due to their 3D design, although wetting may be desirable in some microfluidic strategies [42]. Glass capillary devices have been used in our research group to produce biodegradable polymeric nanoparticles [43,44], liposomes [9], and micelles [45,46] with improved drug loading, encapsulation efficiency, and control over the size distribution. 

In this work, pure or polymer-coated hydrocortisone (HC) nanocrystals have been produced by anti-solvent crystallisation in both co-current flow and counter-current flow focusing glass capillary devices. HC is a poorly water-soluble glucocorticoid drug with an aqueous solubility of 0.31 mg/mL at 25 °C, which is used to treat allergic reactions, autoimmune diseases, skin and lung conditions and certain types of cancers [47,48]. HC is usually administered orally in the forms of tablets and suspensions or topically. When the drug is given orally, only part of the administered dose appears in the plasma. However, HC nanocrystals can be intravenously injected due to their nanoscale dimension, which ensures higher bioavailability and more rapid adsorption of the drug which could be useful in some applications. Surfactants and polymeric stabilisers are commonly used in the synthesis of nanoparticles to ensure their physical stability over time [6,26,49,50]. This work shows the production of size-controlled HC nanocrystals in microfluidic devices, using different polymers and surfactant as stabilisers while adopting the advantages that microfluidic fabrication method and the drug nanocrystal formulation approach provide. High polymer concentrations, however, prevents nanoprecipitation, due to high viscosity of the polymeric solutions, thereby inhibiting the solvent-antisolvent diffusion process [51]. That is why in this work, polymeric stabilisers have not been used directly in the anti-solvent stream, but added in the liquid in the collection vial. Therefore, polymer coating was achieved after collection of the nanocrystals.

## 2. Materials and Methods 

Hydrocortisone (HC) (98% Purity) in powder form was supplied by Fisher Scientific (Loughborough, UK). Hydroxypropyl methylcellulose (HPMC, M_w_ = 86,000 g·mol^−1^) with a viscosity of 2 wt % aqueous solution of 2.6–5.6 Pas at 20 °C was obtained from Sigma-Aldrich (Dorset, UK). Polyvinyl pyrrolidone (PVP, M_w_ = 24,000 g·mol^−1^) was supplied by Sigma-Aldrich. HPMC and PVP served as stabilisers of the produced HC nanocrystals. Sodium dodecyl sulfate (SDS) or sodium lauryl sulfate (SLS) obtained from Fisher Scientific, UK was used as a surfactant to provide additional stabilisation effect. Ethanol used as organic solvent was of analytical grade. The aqueous phase (antisolvent) was distilled water, prepared using reverse osmosis with the aid of Millipore 185 Milli-Q Plus. The organic phase was 7 mg/mL solution of HC in a 60:40 (*v*/*v*) ethanol/water mixture. 

### 2.1. Fabrication of the Glass Capillary Devices

**Figure 1:**
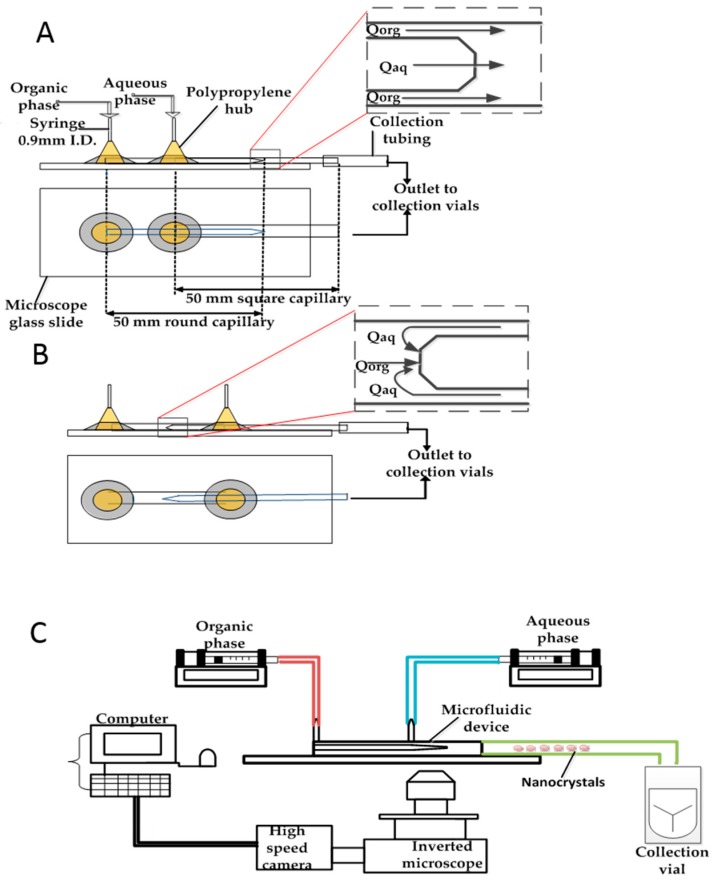
(**A**) Schematic view of the co-flow (CF) glass capillary device; (**B**) schematic view of the counter-current flow (CCF) glass capillary device; (**C**) schematic view of the experimental set up.

The glass capillary microfluidic devices (Figure 1A,B) consisted of a circular glass capillary (Intracel Electrophysiology and Biochemistry Equipment, Saint Ives, UK) which was inserted in a co-axial square glass capillary (Atlantic International Technologies, Rockaway, NJ, USA). The inner (round) capillary had an internal diameter of 0.58 mm and an outer diameter of 1 mm, while the outer (square) capillary had an internal diameter of 1.15 mm and outer diameter of 1.4 mm. The length of both the inner and outer capillaries used was 50 mm. P-97 Flaming /Brown micropipette puller (Sutter Instrument Company, Novato, CA, USA) was used to produce a 20 µm diameter sharp tip of the inner (round) capillary by heating and pulling the capillary for about 20 s at a pre-determined setting. The nozzle diameter (orifice diameter) was then adjusted by grazing, using sandpaper (Black Ice Waterproof T402 Paper, Alpine Abrasives, Leicester, UK) until the desired orifice diameter was achieved and the orifice had a smooth edge. Microforge MF-830 (Intracel Electrophysiology and Biochemistry Equipment) microscope was used to confirm the size of the orifice of the inner capillary. The inner (round) capillary was then carefully and centrally placed in the outer (square) capillary before two syringe needles (2.5 mm outer diameter and 0.9 mm inner diameter, B-D Precisionglide^®^, Sigma-Aldrich) with plastic hubs were placed at strategic locations on the device. A two-component epoxy glue (Five Minute^®^ Epoxy, ITW Devcon Limited, Shannon, Ireland) was used to hold all the components in place on a microscope slide (Sigma-Aldrich). The entrances of the organic and aqueous phase were situated inside the plastic hubs and both liquids were delivered to the capillaries through polyethylene medical tubes (0.86 mm inner diameter and 1.52 mm outer diameter, Smiths Medical International Limited, Ashford, UK), via the syringe needles. A collection tube (1.58 mm inner diameter and 2.1 mm outer diameter, Sigma-Aldrich) was attached to the tip of the square capillary (for the co-current device) and round capillary (for the counter-current device) to collect the product. 

### 2.2. Set up and Preparation of Nanocrystals

The generation of nanocrystals using the fabricated microfluidic device was achieved by the set up shown in Figure 1C. Two SGE syringes were fixed to two 11 Elite syringe pumps (Harvard Apparatus, Cambridge, UK) to deliver the organic phase and the aqueous phase to the inner capillary and outer capillary respectively. The microfluidic device was mounted on the GXM XD63 optical inverted microscope (GX Microscopes, Hagerstown, MD, USA). The formation of nanocrystals occurred in the device and was monitored with the Phantom V9.0 high-speed camera (Vision Research, Ametek, Berwyn, PA, USA) connected to the inverted microscope, to capture and record the video of the process at 25 frames per second and a resolution of 576 by 288 pixels. 

The organic phase for all experiments was prepared by dissolving 70 mg of HC in 10 mL of a 60:40 (*v*/*v*) ethanol:water mixture. The organic and aqueous phases were introduced to the reactor at different flow rates and mixed together in the mixing zone downstream from the orifice to form the nanocrystals in the outer capillary (for CF device) or the inner capillary (for CCF device). The produced nanosuspensions were introduced into a continuously stirred mixture of stabilisers added in the collection vial. The coating mixture was composed of PVP (0–0.2 g/mL), SDS (0–0.05 g/mL), and HPMC (0–0.2 g/mL) in various combinations. To investigate the effect of the device geometry, CF and counter-current flow (CCF) devices were fabricated with the nozzle diameter of 50 µm or 100 µm. The flow rate of the organic phase in these experiments was kept at 1.5 mL/h while the aqueous phase flow rate was varied from 7.5 mL/h to 37.5 mL/h to obtain the volume ratio of the organic phase to aqueous phase of 5 to 25 (Section 3.4.1). The effect of HPMC was investigated in a CF device using the volume ratio of the organic phase to aqueous phase of 1 to 5 (Section 3.1). The prepared nanocrystals were sonicated for 15–20 min using Ultrasonic bath (FB 15046, Fisher Scientific, Loughborough, UK) at mild power to coat the stabilisers and then centrifuge, using Hermle Z 383 K centrifuge (Hermle Labortechnik, Wehingen, Germany), at a speed of 15,000 rpm for 80 min. After centrifugation, the clear liquid was removed while the residue was washed to remove as much solvent from the sample as possible. The centrifugation was repeated four times, while adding more distilled water to the residue after each cycle. The resultant sample, almost completely free from solvent was then frozen overnight in a vial to prepare for freeze drying, and obtain the solid sample of the processed hydrocortisone.

### 2.3. Solubility of Hydrocortisone in Ethanol/Water Mixtures

**Figure 2:**
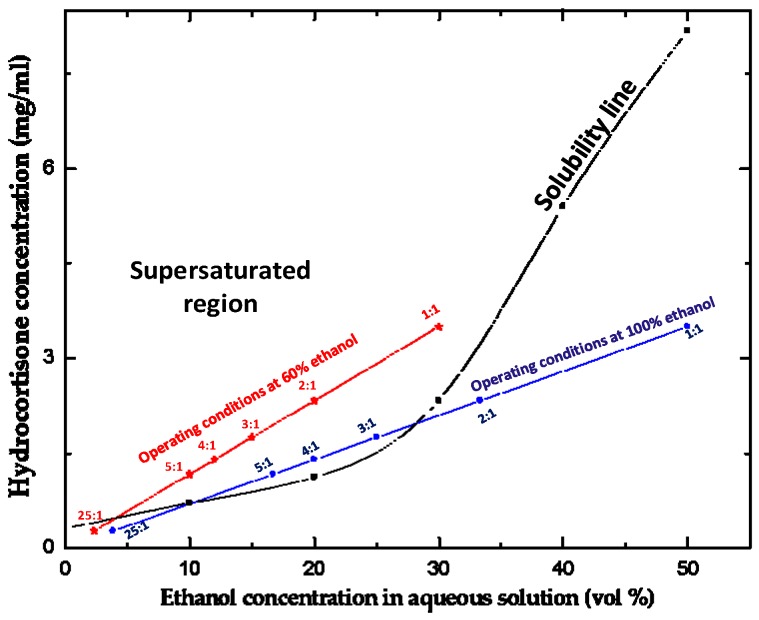
The solubility curve of hydrocortisone (HC) at 298 K [52] and two operating lines indicating the HC and ethanol concentrations in the collection tube after mixing of the two phases at the flow rate ratio of the aqueous to organic phase ranging from 1:1 to 25:1. The concentration of HC in the incoming organic phase was 7 mg/mL at all mixing ratios.

The solubility curve for HC in water/ethanol mixtures at 298 K [52] was compared with two operating lines constructed for the organic phase composed of 100% ethanol or a 60:40 (*v*/*v*) mixture of ethanol and water. The operating line (Figure 2) indicates the drug and ethanol concentrations in the collection tube after complete mixing of the two phases, but before formation of any particles. In each case, the initial HC concentration in the organic phase was 7 mg/mL and the aqueous to organic phase volume ratio ranged from 1:1 to 5:1. 

HC is completely soluble in 100% ethanol, but the solubility in pure water at 298 K is only 0.31 mg/mL, which means that the HC solubility in ethanol-water mixture shows large swings from nearly zero for pure water to infinity for pure ethanol. For crystallisation of HC to occur in the collection capillary, the operating line must be above the solubility line, e.g., in the supersaturated region of Figure 2. For pure ethanol, the operating line is below the solubility line at the aqueous to organic mixing ratios of 1:1 and 2:1 due to high concentration of ethanol in the mixed stream. HC nanocrystals cannot be formed under these conditions since the resultant solution is unsaturated. At *Q*_aq_:*Q*_org_ of 3:1, 4:1 and 5:1, the operating line for 100% ethanol is above the saturation line, but the gap between the two lines is very small. Therefore, the yield of nanocrystals would be rather low if 100% ethanol is used as solvent. In order to obtain a reasonable yield of drug nanocrystals, HC was dissolved in a 60/40 mixture of ethanol and water, rather than in pure ethanol. In this case, the operating line was above the solubility line for all aqueous to organic mixing ratios except 25:1. 

### 2.4. Characterisation of Produced Nanocrystals (Quantitative and Qualitative Analyses)

The HC nanocrystals produced during the experiments undertaken were characterised to determine their size, morphology, inner structure and stability. The particle size analysis of the nanocrystals produced was carried out using a Delsa^TM^ Nano Particle Analyzer (Beckman Coulter, Inc., Brea, CA, USA). The size of nanoparticles produced, especially in drug delivery, is important because it has a profound role in controlling the cellular fate and biodistribution of the nanoparticles [53].

#### 2.4.1. Particle Size Analysis

Delsa^TM^ Nano Particle Analyzer uses dynamic light scattering technique by measuring the fluctuations of light as a function of time. This is achieved by a laser diode which emits light at a wavelength of 658 nm directed at the bottom part of the cuvette containing sample to be analysed. The light is scattered at different directions on hitting the sample, but a detector is placed at an angle of 165° from the source of the light to measure back-scattered light at the same angle.

The sample was placed in a 4-mL disposable cuvette with measurements taken three times at a temperature of 25 °C and for 70 different readings for each run. The cuvette was placed inside the Delsa^TM^ Nano chamber, strategically designed to ensure that 3 different planes (*x*-, *y*- and *z*-plane) are observed to give a more accurate analysis of the sample. The equipment uses CONTIN method of analysis to calculate the *Z*-average mean, which is the harmonic intensity averaged particle diameter. The CONTIN algorithm method uses the relative intensities detected against the size of the particles that scattered them together with the autocorrelation function for analysis. Another method of analysis carried out by the equipment is the cumulants analysis. This analyses the autocorrelation function and produces a mean value for the size, *Z*-average, which is an intensity-based value, and the polydispersity index (PDI), which is a dimensionless measure of the broadness of the size distribution.

#### 2.4.2. Differential Scanning Calorimetry (DSC) Analysis

The analysis was carried out using a Differential Scanning Calorimeter, Q10 (TA Instruments, New Castle, DE, USA) at a scanning rate of 10 °C/min and temperature range of 20 to 300 °C. Each sample, weighing between 4 mg and 10 mg was put in a sealed aluminium pan, with an empty pan used as reference. The differential scanning calorimeter shows the thermal profile of the sample by indicating the heat flow required to increase the temperature of the sample. The thermal profile of both the unprocessed and processed drug was compared. The degree of crystallinity was calculated using the measured heat of fusion and the enthalpy of cold crystallization as given in literature [54].

#### 2.4.3. X-ray Powder Diffraction (XRPD) Analysis

The crystallinity of the sample was assessed using the D2 Phaser X-ray powder diffraction (XRPD) diffractometer (Bruker Corporation, Billerica, MA, USA). The unprocessed drug was checked on the X-ray powder diffractometer and the result obtained was compared with the International Centre for Diffraction Data (ICDD) reference database PDF-2. The produced and dried nanocrystals were also analysed and compared to the unprocessed sample. This is necessary as purity matters in nanoscale level, to ensure the drug was sufficiently pure. The samples were scanned over an angular range of 2°–50° 2θ, with a step size of 0.02°, a rotation speed of 15 rpm, and a count time of 1 s per step.

**Figure 3:**
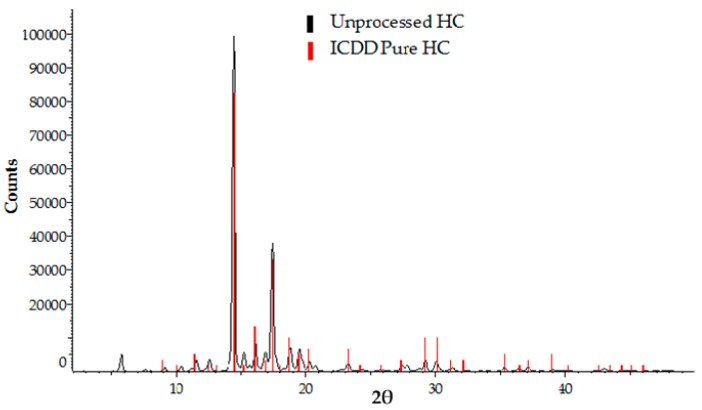
X-ray powder diffraction (XRPD) profiles of unprocessed hydrocortisone compared to an XRPD standard for HC structure Joint Committee on Powder Diffraction Standards (JCPDS) reference number 00-015-1016 in the International Centre for Diffraction Data (ICDD) database.

The peaks obtained for the unprocessed hydrocortisone shown in Figure 3 corresponded to most of the peaks from the ICDD database for pure hydrocortisone meaning that the drug was pure and the XRPD method was reliable.

#### 2.4.4. Transmission Electron Microscopy (TEM)

The transmission electron microscopy (TEM) analysis was done by placing a drop of the nanosuspension on carbon-coated mesh and drying the sample in the air. The analysis was done on a JEOL JEM-2000FX analytical TEM which was operated at the voltage of 200 kV.

## 3. Results and Discussion

**Figure 4:**
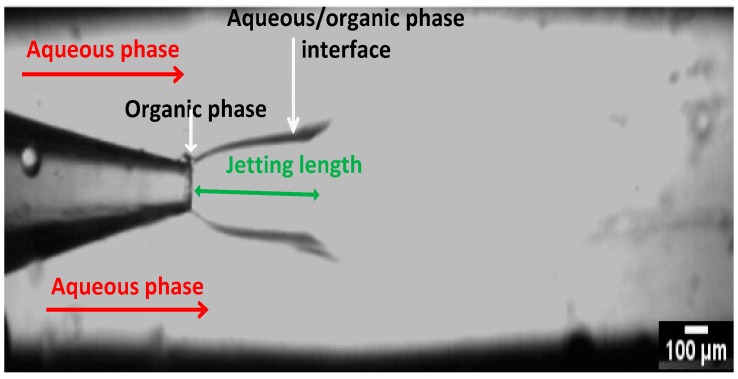
A CF glass capillary device with an orifice diameter of 100 µm operating at the flow rates of the aqueous phase of 7.5 mL/h and the organic phase of 1.5 mL/h. The organic phase contained 7 mg/mL of HC in a 60:40 (*v*/*v*) ethanol:water mixture and the aqueous phase was Milli-Q water.

The micrograph in Figure 4 shows the micromixing in the CF device with an orifice diameter of 100 μm during production of the nanocrystals. The equilibrium interfacial tension at water/ethanol interface should be zero as ethanol and water are miscible in all proportions. However, when two miscible fluids are brought in contact, initial gradient of concentration at the boundary can give rise to temporary tension between the fluids, known as the transient interfacial tension or the Korteweg stress. The transient interfacial tension at any point is proportional to the product Δ*C*^2^/δ, where ΔC is the difference in ethanol concentration in the boundary region and δ is the boundary region thickness. The transient interfacial tension changes with time in proportion to (*D*/*t*) ^0.5^, where *D* is the diffusion coefficient of ethanol and *t* is the interfacial age. In a continuous microfluidic device, the interfacial age is constant at any location on the interface, but varies along the interface from one location to another. In fact, the interfacial age t, has the minimum value at the orifice and increases in the downstream direction until the transient interfacial tension becomes so low that the interface can no longer persist. In Figure 4, the water/ethanol interface disappears at the distance of approximately 5 orifice diameters downstream of the orifice. The interface is elongated because the transient interfacial tension is overcome by the inertia of the organic stream. At low organic stream velocities, the interface was found to be spherical since the transient interfacial tension dominated over inertial forces [43]. Nanocrystals were predominantly formed at the interfacial area due to diffusional exchange of water and ethanol, leading to supersaturation. The jet has a widening cross section, reflecting the fact that the organic phase velocity at the inlet section was 0.13 m/s, and the aqueous phase velocity was 0.0016 m/s. The difference in fluid velocities has led to gradual decrease in the organic phase velocity and corresponding increase in the aqueous phase velocity until a steady-state was eventually established with the parabolic velocity profile over the whole cross section.

### 3.1. Effect of HPMC on the Particle Size of the Nanocrystals

The stability of the produced nanocrystals was achieved by collecting the nanodispersion in a stirred vial containing either a mixture of PVP and SLS or a mixture of HPMC, PVP, and SLS. HPMC, also known as hypromellose, is chemically modified cellulose, added as an enteric coating to protect hydrocortisone from premature degradation by gastric acid in the gastrointestinal tract. HPMC is a biodegradable and biocompatible excipient, generally recognized as safe by the US Food and Drug Administration (FDA). It is widely used in pharmaceutical formulations [55,56].

**Figure 5:**
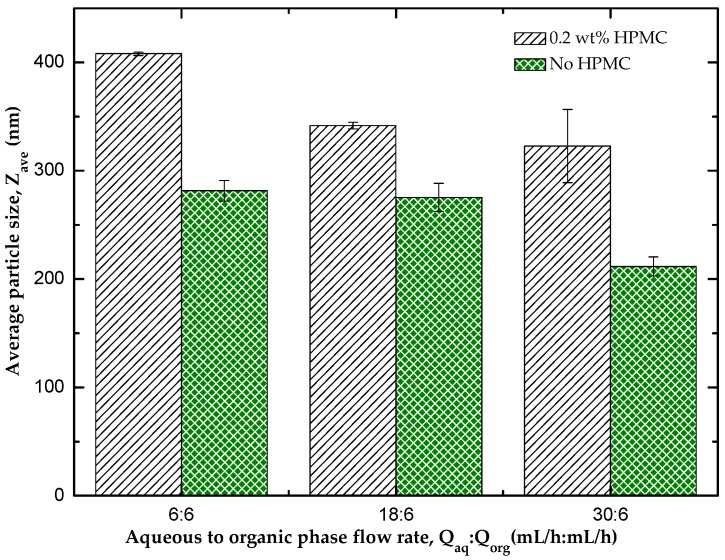
The effect of hydroxypropyl methylcellulose (HPMC) coating on the average size of HC nanocrystals produced using a CF device with an orifice diameter of 100 μm. The nanocrystals were collected in an aqueous solution containing 0.2 wt % polyvinyl pyrrolidone (PVP) and 0.05 wt % sodium lauryl sulfate (SLS) with and without the addition of 0.2 wt % HPMC. The flow rate of the organic phase was kept constant at 6 mL/h, while the flow rate of the aqueous phase was varied from 6 mL/h to 30 mL/h.

Figure 5 shows the average diameter of the nanocrystals coated with a mixture of HPMC, PVP and SLS and those stabilized only by PVP and SLS. It was observed that the size of nanocrystals for the samples prepared without HPMC was significantly lower than the size of nanocrystals in which HPMC was used as part of the stabilisers. This can be explained by the steric effect of the cellulose forming a protective layer around the nanocrystals synthesised [6], thereby increasing the overall size of the nanocrystals, although this layer inhibits growth of the nanocrystals. The size of the nanocrystals decreased with increasing *Q*_aq_/*Q*_org_ ratio, but this effect is discussed in more details in Section 3.4. 

### 3.2. Analysis of Crystallinity of Hydrocortisone Nanocrystals

The degree of crystallinity of raw HC powder was compared with the crystallinity of the dried samples produced in the microfluidic device and coated with different stabilisers. It is important because the same drug dispersions with different degree of crystallinity exhibit different physical and chemical stability, saturation solubility, and dissolution rate, which may significantly impact drug bioavailability and its biodistribution after administration. The degree of crystallinity also affects physical properties of the solid drug, such as thermal, electrical and optical properties [57].

#### 3.2.1. Differential Scanning Calorimetry (DSC) Analysis

**Figure 6:**
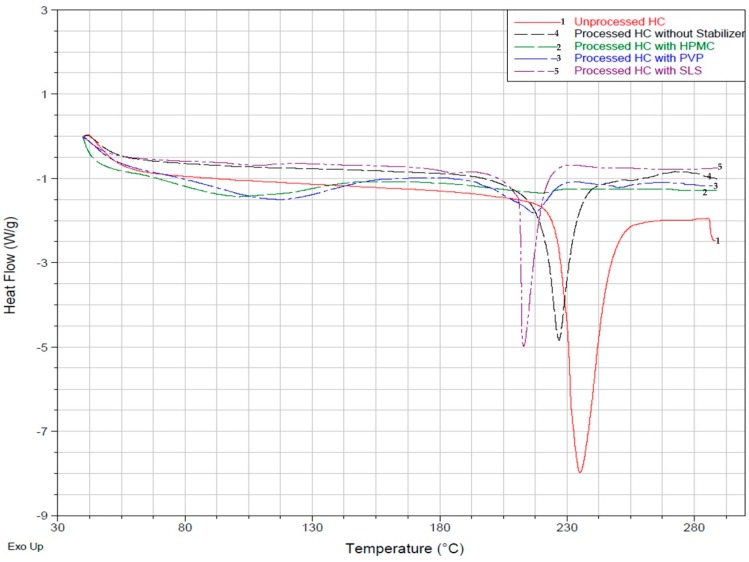
Overlay of the differential scanning calorimetry (DSC) thermal profiles of the unprocessed HC and processed (precipitated) HC in 100 μm counter-current flow (CCF) microfluidic device at aqueous phase and organic phase flow rates of 6 mL/h. The stabilisers used independently were 0.2 wt % HPMC, 0.2 wt % PVP, and 0.05 wt % SLS.

Differential scanning calorimetry (DSC) thermal profile was analysed for the unprocessed HC and the processed HC coated with different polymers and/or surfactants. The peak of the unprocessed HC sample (Line 1 in Figure 6) occurs at 235 °C, corresponding approximately to the melting point of the pure drug. The peaks of all processed (precipitated) drug samples were considerably smaller and shifted to lower temperatures. This suggests a decrease in crystallinity of HC after nanoprecipitation and downstream processing of the nanosuspension produced in the device. This reflects an important role of polymeric stabilisers and surfactants on the physical state of the processed drug.

The DSC curve of the sample prepared without the use of any stabiliser (Line 4) shows a significantly smaller peak occurring at 227 °C. The degree of crystallinity of 65% was smaller than in the unprocessed drug due to imperfections in the crystal lattice caused by rapid precipitation of the drug. The amorphous form, because of its thermodynamic properties is less stable and is able to undergo phase transformation to a stable crystalline form. As mentioned previously, the amorphous form is more soluble in water than the crystalline form and shows higher dissolution rates. When SLS was used as a stabilizer (Line 5), the peak shifted farther to lower temperatures and the degree of crystallinity was 44%. SLS is an anionic surfactant which ensures repulsion between the drug nanocrystals and ensures agglomeration is kept low during storage.

The degree of crystallinity of the sample containing HPMC as the only stabiliser was only 3%. Two small peaks were observed on the DSC curve 2: an almost negligible peak at 217 °C corresponds to the melting point of the drug and a broad peak at 105 °C corresponds to the glass transition temperature *T*_g_ of HPMC. The *T*_g_ value of pure HPMC is 162–180 °C [58]. Mixing of a drug that has a low *T*_g_ with a high *T*_g_ polymer at the molecular level leads to the development of a system with *T*_g_ intermediate to these two components. Hence, the *T*_g_ value of the drug increases and it undergoes anti-plasticization, whereas the *T*_g_ value of HPMC decreases and it undergoes plasticization. A significant decrease in *T*_g_ for HPMC in the drug formulation compared with pure HPMC indicate that strong molecular interactions exist between the drug and HPMC. Several studies have shown the formation of ion-dipole interactions and intermolecular H-bonding between various drugs and polymers. PVP showed a relatively higher degree of crystallinity of 20% when used as the only stabiliser in the system when compared to HPMC. PVP is an anionic surfactant which provides kinetic stability of the nanocrystals, but also improves thermal stability of the drug. As a conclusion, the degree of crystallinity in various samples changes in the following order: HPMC < PVP < Combination of all stabilisers < No stabiliser < Unprocessed material.

#### 3.2.2. X-ray Powder Diffraction (XRPD) Analysis

**Figure 7:**
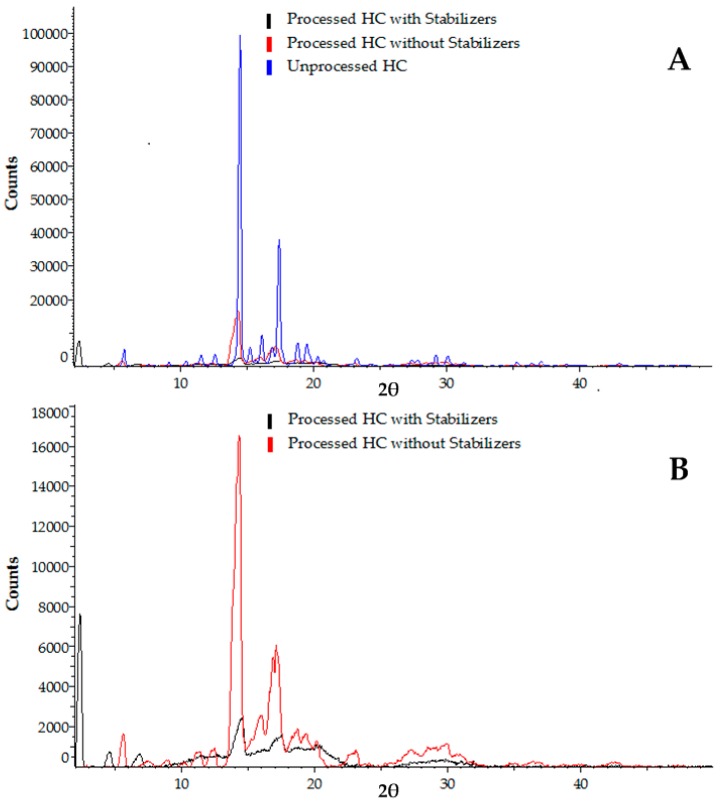
XRPD profiles: (**A**) the unprocessed HC powder compared with processed HC samples prepared with and without stabilisers; (**B**) the processed HC samples prepared with and without stabilisers. The stabilisers included in the mixture are 0.2 wt % HPMC, 0.2 wt % PVP, and 0.05 wt % SLS. The CF microfluidic device with an orifice diameter of 100 μm was used. The flow rates of 6 mL/h were used for both the organic phase and aqueous phase.

The processed HC samples were analysed and the obtained XRPD patterns were compared with the pattern of the unprocessed drug to observe the changes in crystallinity, as shown in Figure 7A. The unprocessed and processed drug samples were scanned using the same settings, i.e., an angular range of 2°–50° 2θ, a step size of 0.02° and 1 s per step. 

Most of the peaks observed in the profile of the unprocessed drug shown in Figure 7A disappeared or were reduced considerably in the profiles for the processed drug samples, which confirms the changes in crystallinity of the HC, suggesting transformation to an amorphous solid state of the drug in the processed sample. It can be attributed to the rapid drug precipitation in the device, but the downstream processes (freeze drying, centrifugation, temperature changes, etc.) have contributed to this change as well [6,57].

The peak intensities in the XRPD pattern of the sample produced with the addition of stabilisers were reduced considerably compared with the sample prepared without any stabiliser, as shown in Figure 7B. It shows a significant effect of the stabilisers on the crystallinity of the drug samples. 

### 3.3. Morphology of the Produced Nanocrystals

**Figure 8:**
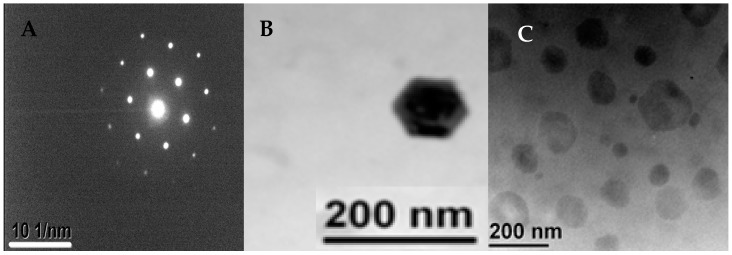
TEM images of processed HC in the microfluidic device: (**A**) the crystal structure of the processed HC (**B**) showing the hexagonal crystal structure of the drug encapsulated within the stabilisers; (**C**) showing polymeric micelles around the drug nanocrystals. A CCF microfluidic device with an orifice diameter of 200 μm was used to produce the nanocrystals, with an aqueous phase flow rate of 22.5 mL/h and an organic phase flow rate of 1.5 mL/h. The stabilisers include 0.2 wt % HPMC, 0.2 wt % PVP, and 0.05 wt % SLS.

The processed drug as shown in the TEM images of Figure 8A–C shows the drug crystals and the micelles formed by the stabilisers around the drug. The sizes shown are in correlation with the values obtained from the particle size analyses of the sample. The crystal structure of HC drug varies depending on the solvent used [59].

### 3.4. The Effect of Operating Parameters and Geometry on the Size of Nanocrystals 

#### 3.4.1. Counter Current vs. Co-current Flow

Counter current (CCF) and co-current flow (CF) devices with two different orifice sizes (50 μm and 100 μm) were used to produce HC nanosuspensions by mixing the organic phase at 1.5 mL/h and the aqueous phase at 7.5–37.5 mL/h. No stabilisers were added in the collection vial. 

**Figure 9:**
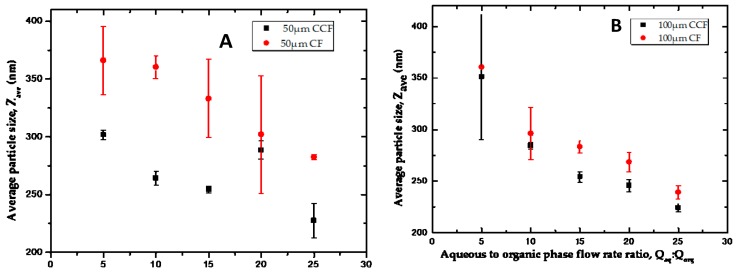
The Average particle size of HC nanocrystals prepared using CCF or CF device with an orifice size of: (**A**) 50 µm; (**B**) 100 µm. No stabilisers were added in the collection vial as the samples were analysed immediately after production. The organic phase flow rate was held constant at 1.5 mL/h, while the aqueous phase flow rate was varied to obtain different flow rate ratios.

**Figure 10:**
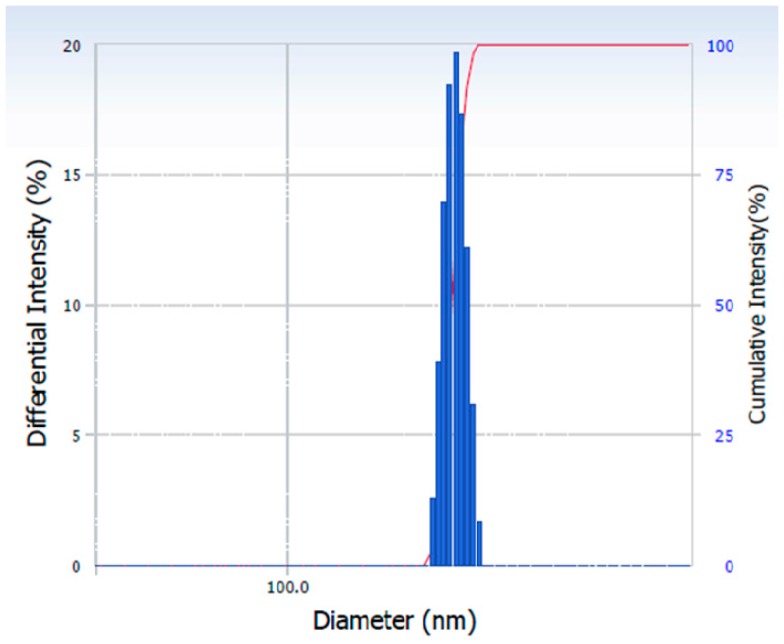
The particle size distribution of HC nanocrystals prepared using CF device with an orifice size of 200 µm. No stabilisers were added in the collection vial as the samples were analysed immediately after production. The organic phase flow rate was 1.5 mL/h, while the aqueous phase flow rate was 30 mL/h. The size of nanoparticles measured was 226 ± 12 nm.

The aqueous phase flow rates were varied to obtain flow rate ratios, *Q*_aq_:*Q*_org_, of 5, 10, 15, 20, and 25 and three different runs were carried out for each condition. As shown in Figure 9, for each orifice size the nanocrystals produced in the CCF device were smaller than those obtained in the CF device. This can be explained by the constriction of the inner capillary affecting the micromixing process in the CCF device, as shown in Figure 10. It is also due to higher shear stresses at the point of mixing of the two phases due to their counter-current flow [10,60]. The smaller particle size in the CCF device may also be explained by the reduced mixing time of the organic phase and the antisolvent. The average time *t* for a molecule to diffuse over a distance x is given by: *t* = *x*^2^/(2*D*), where *D* is the diffusion coefficient. The nanoprecipitation occurs at the aqueous/organic phase interface and the maximum distance each drug molecule must travel to reach the interface equals the width of the organic phase stream. In a CCF device, the width of the organic phase jet is smaller than the orifice size, while in the CF device, the width of the organic phase stream is generally greater than the orifice size. A lower width of the organic phase stream leads to shorter mixing times and smaller particle sizes in the CCF devices under the same flow rates. A lower mixing time than induction time of crystallization is essential to generate uniformly-sized nanocrystals [61]. 

The particle size distribution of the HC nanocrystals produced is shown in Figure 10, while the polydispersity index (PDI) (Table 1) of the samples confirms the production of monodispersed HC nanocrystals as expected with the use of microfluidic devices.

#### 3.4.2. The Effect of Flow Rate Ratio

The HC nanocrystals obtained shows a downward trend with increasing flow rate ratio, and an increase in aqueous phase flow rate led to a decrease in the size of the nanocrystals produced. This was in agreement with what was reported [6,9,62], where increase in the flow rate of the antisolvent while the organic phase remained constant produced smaller mean particle size of the product. This can be explained by the fact that the size of the nanocrystals is determined by the relative rates of nucleation stage, crystal growth, and aggregation. The shorter mixing time favours nucleation over crystal growth, which should lead to smaller average particle size, since larger amount of smaller crystals will be formed. It can also be explained by the increasing Reynolds number as a result of higher flow rates improving the mixing within the system [6,61]. Finally, the agglomeration of formed nanocrystals is less likely at *Q*_aq_/*Q*_org_ ratios, due to higher dilution factors of the drug in the formed nanosuspension. The rate of aggregation of the nanocrystals is proportional to their collision rate, which is lower in more diluted suspensions. 

#### 3.4.3. The Effect of Device Geometry 

In all the devices, it was observed that the higher the volume ratio, the smaller the nanocrystals produced. The microfluidic devices with 200 µm orifice diameter produced smallest nanocrystals while the device with the nozzle diameter of 400 µm produced the largest nanocrystals (the results are not shown here). Therefore, the orifice size of the inner capillaries of the devices influences the size of nanocrystals produced. This is in agreement with what was reported in formation of lipid vesicles using microfluidic flow-focusing device, where it was noted that channel diameter affects the size of vesicles produced [63].

**Figure 11:**
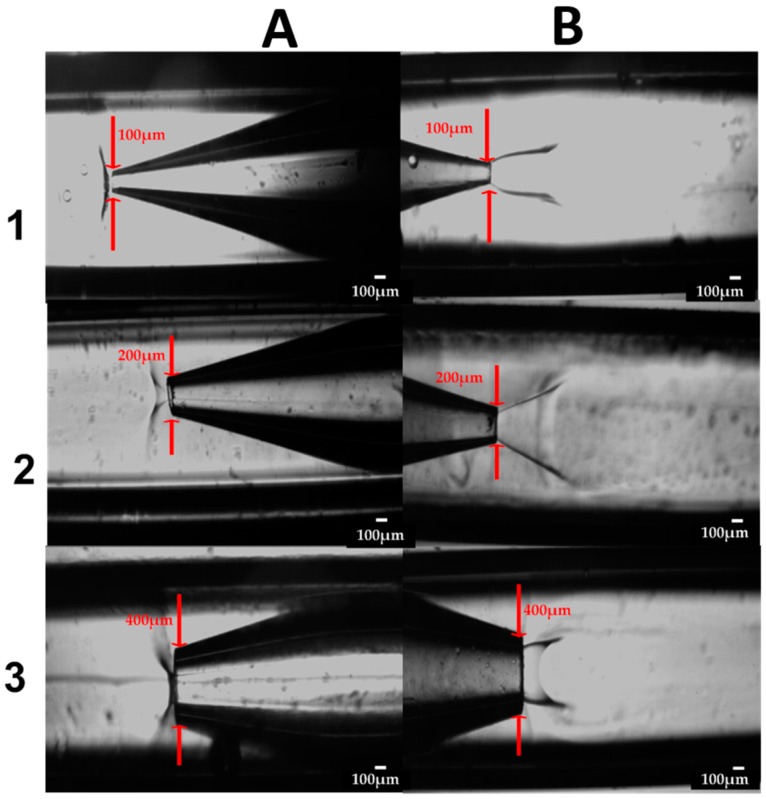
Micrographs showing the drug nanoprecipitation in glass capillary devices: (**A**) CCF device; (**B**) CF device; 1, 2 and 3 represents the 100 µm, 200 µm, and 400 µm devices, respectively. The flow rates of the aqueous and organic phase were 7.5 and 1.5 mL/h, respectively.

The micrographs in Figure 11 show the regions where micromixing occurs in both the CF and CCF microfluidic devices for nozzle diameter of 100 μm, 200 μm and 400 μm. The jetting lengths are longer for the CCF device in comparison to the CF devices, although in all CF devices, the transient interfacial tension is more prominent than in CCF devices for the same conditions. In other words, the boundary line between the organic and aqueous stream in the outer capillaries is much sharper than the boundary between the organic and aqueous stream in the inner capillaries reflecting the fact that the CCF devices provide more efficient micro-mixing than the CF devices. This is because the point of contact of the two streams within the CF device occurs were the fluid velocities are relatively small due to large cross-sectional area, unlike the CCF device [43]. The jet lengths in the CF devices were longer for the inner capillaries with larger orifice; the minimum jet length was obtained at the orifice diameter of 400 μm due to the lowest velocity of the organic phase. The deposition of the formed nanocrystals onto the outer wall of the injection capillary was observed in all CF devices, while inner capillaries of the CCF devices were relatively free from the deposited crystals. It should be noted that in CF devices, a big difference in velocities of the organic phase and aqueous phase near the injection point leads to vortex flow in the aqueous phase that can bring and deposit the nanocrystals upstream of the orifice. 

## 4. Conclusions 

Hydrocortisone (HC) nanocrystals coated with hydroxypropyl methylcellulose (HPMC), polyvinyl pyrrolidone (PVP) and sodium lauryl sulfate (SLS) were produced by nanoprecipitation in coaxial assemblies of glass capillaries using both co-flow (CF) and counter-current flow (CCF) of the organic and aqueous phase. The size of the nanocrystals decreased with increasing aqueous to organic flow rate ratio from 5 to 25 and the total liquid flow rate in the collection capillary. The produced nanocrystals were coated in a stirred vial with a mixture of surfactants and polymeric stabilisers. The size of drug nanocrystals increased significantly after coating with 0.2 wt % HPMC, due to steric effects. The CCF microfluidic devices provided smaller nanocrystals than the CF devices under the same flow rates and for the same orifice size of the inner capillary, due to more efficient mixing. In addition, deposition of nanocrystals onto the walls of the inner capillary was less pronounced in CCF devices. A transient interfacial tension was observed when the two liquids were brought in contact due to high concentration and density gradients at the boundary between the two phases but quickly decayed to zero due to solvent displacement at the interface. 

The stabiliser used to encapsulate the hydrocortisone nanocrystals had a huge effect on the physical properties of the nanoparticles, such as the degree of crystallinity and melting point. The amorphous content of the drug nanocrystals composed of 100% drug was smaller than that of the unprocessed (raw) drug powder, due to rapid precipitation in the microfluidic device and the effect of downstream processing such as freeze-drying. The amorphous form of the drug can be useful in providing higher solubility and dissolution rate, and thus improved drug bioavailability after oral intake. The degree of crystallinity of the dried nanosuspensions varied depending on the type of coating in the following order: HPMC < PVP < SLS < No stabiliser < Unprocessed drug powder. The degree of crystallinity of the drug stabilized with HPMC was only 3%, which means that the drug was dispersed in HPMC matrix in the form of molecular dispersion or amorphous domains. The XRPD analysis confirmed the changes in the crystallinity on the precipitated drug samples. The peaks, especially the ones obtained at 2θ of 6°, 14.5°, 17°, 18°, and 19° for the unprocessed drug, were significantly reduced in the processed hydrocortisone drug, especially in the absence of stabilisers. 

## Figures and Tables

**Table 1:** The polydispersity index (PDI) of HC samples produced in CF microfluidic devices with 50 μm and 100 μm orifice sizes, showing the monodispersity of nanocrystals produced.

Flow Rates (*Q*_aq_:*Q*_org_, mL/h:mL/h)	50 μm Orifice Device	100 μm Orifice Device
	PDI	PDI
6:6	0.253 ± 0.005	0.236 ± 0.039
18:6	0.405 ± 0.038	0.203 ± 0.038
30:6	0.612 ± 0.086	0.327 ± 0.023
